# Supplementary material for: Predicting ruminal degradability and chemical composition of corn silage using near-infrared spectroscopy and multivariate regression
Source: PLoS One. 2024 Apr 18;19(4):e0296447. doi: 10.1371/journal.pone.0296447 (PMC11025743; doi:10.1371/journal.pone.0296447)
Supplement: Attachment — Submitted filename: Response to Reviewers.docx [file pone.0296447.s004.docx]

October 10th, 2023 – Fargo, ND, USA

Manuscript Number: PONE-D-23-18553

Title: Predicting ruminal degradability and chemical composition of corn silage using near-infrared spectroscopy and multivariate regression

Dear Section Editor Aziz ur Rahman Muhammad,

We are submitting a revised version of the manuscript number PONE-D-23-18553 “Predicting ruminal degradability and chemical composition of corn silage using near-infrared spectroscopy and multivariate regression”. We would like to thank you for the opportunity to revise and improve the manuscript. Also, we thank the editor and reviewers for the important contributions to our manuscript.

We have tried to incorporate the suggestions as much as we could. All modifications and replies of questions or concerns are detailed described below. In the file ‘Revised Manuscript with Track Changes’, the changes are in green (reviewer #1), red (reviewer #2), and blue (reviewer #3) colors to facilitate the revision process.

Please, let us know if you have any additional comments or criticism. We look forward to your positive feedback on this revised version.

Thanks, in advance!

Sincerely,

The authors.

**Replies to Reviewer #1**

**Reviewer #1: The paper addresses an interesting issue, the use of NIR, which represents an interesting, low-impact and very rapid precision technology. Indeed, I do not find correct to use the validation of *in vivo* degradability with a very low number of samples (about 20).**

**I suggest to modify the paper without the in vivo test. The authors could divide it into 2 different papers.**

AU: Thank you for your careful and constructive criticisms regarding our manuscript. We appreciate your thoughtful consideration of our work. Regarding the *in vivo* degradability validation, we would like to clarify that our study examined *in situ* degradability evaluation, which is the most common approach for measuring nutrient degradability by researchers. Our objective was to explore the possibility of developing predictive models given the inherent challenges in obtaining *in situ* degradability parameters, including the need for cannulated animals and the substantial number of samples to be processed and analyzed. Despite the limited sample size, we implemented cross-validation to enhance the reliability of our findings. In this manner, even with the constrained number of samples, we can gain insights into the feasibility of obtaining *in situ* degradability parameters using NIR spectroscopy. This insight could guide future studies. We had pointed out in the discussion and conclusion that larger experiments are needed to improve the prediction models.

We believe that presenting both the *in situ* evaluation and chemical composition analysis in a single paper allows for a more comprehensive nutritional assessment of using NIR spectroscopy for evaluating corn silage.

**Other important issues:**

**Line 80- Sample collection and preparation**

**Add information concerning:**

**-how much samples have been collected?**

**-time of collection**

**-which corn hybrid**

**-climate characteristics of the samples site**

AU: We collected a total of 94 samples, all of which were evaluated. However, the specifics you've mentioned, such as the exact time of collection, corn hybrid details, and precise climate characteristics, unfortunately, aren't available due to the nature of the sample collection process, which involved collaboration with rural producers and technical consultants, who did not have precise control over these factors during sample collection. Also, our objectives were not to examine different hybrids, but were to examine feed stuffs with different nutrient composition and degradability. We appreciate your understanding and remain available for any further inquiries.

**Line 88-I don’t understand the numbers of analyzed samples. 94 samples were analyzed with NIRS but only 23 were tested in vivo. Please explain that.**

AU: Thank you for your question regarding the number of analyzed samples. We indeed assessed all 94 samples for their chemical composition using NIRS. However, only 23 of them were subjected to *in situ* degradability assessment. This limitation can be attributed to various factors. Firstly, the quantity of samples provided by our collaborators was constrained, as *in situ* evaluation requires a larger sample volume. Additionally, our ability to evaluate more samples was restricted due to resource limitations at that time. These limitations included the availability of cannulated animals and the labor required to conduct the *in situ* assays. We hope this provides clarity, and we appreciate your valuable inquiry.

**I suggest to compare the NIR spectra presented in this paper with that one published in the article Zicarelli, F.; et al. Agronomy 2023, 13, 634. https://doi.org/10.3390/ agronomy13030634**

AU: Thank you for your suggestion. However, it appears that the referenced paper does not provide the NIR spectra used in their study. Instead, we utilized the prediction parameters from the models presented in the article you suggested, as the NIR spectra data was not available. Please, check them on lines 309-318. If there are any additional sources or data you recommend for comparison, we would be more than happy to consider them.

**Replies to Reviewer #2**

**Reviewer #2: The authors have performed the analysis in a scientific manner and presented it in a wholely defined manner. I highly recommend the acceptance of the manuscript with minor revision.**

AU: Thank you very much for your positive evaluation and recommendations. We are grateful for your encouraging feedback and recommendation for the acceptance of the manuscript with minor revisions. Your insights have been invaluable.

1. **Kennard-stone algorithm is utilized for dataset classification. Please add few advantages** **and limitations of the algorithm.**

Thank you for the valuable suggestion. We have now included a description of the Kennard-Stone algorithm in the Methods section of the paper, outlining its advantages. Specifically, we have included information on the advantages of the Kennard-Stone algorithm, which can be found in lines 188-192 of the revised manuscript.

2. **Please explain concordance co-efficient.**

We have described in the text the meaning of the CCC and the reference for the reader to go if they want more information.

**I have no major issues prior submission to the manuscript except above stated comments.**

**Replies to Reviewer #3**

**Reviewer #3: General**

**This manuscript needs some revision before suitable for publication and the overall structure needs to be revised. The authors do need to provide further justification of the study as some similar studies have been published but the authors have not cited these in the introduction and identified the new information provided by their work. The novel aspects of the work need to be clearly identified.**

**The number of abbreviations in the manuscript is excessive and reduces the overall readability. The number of abbreviations needs to be reduced.**

**A combined results and discussion section can be difficult to read, I would suggest that the results and discussions sections are separated.**

AU: Thank you for your valuable feedback and constructive suggestions. We greatly appreciate your time and effort in reviewing our manuscript. Regarding the introduction, we agree with your comments. We have revised the manuscript and incorporated additional information to provide further justification for our study. Please refer to the introduction, specifically lines 72-76. Additionally, we've also made adjustments to the objectives of the study to ensure alignment with your suggestions. The updated objectives can be found in lines 79 and 80.

In response to the concern about the excessive use of abbreviations, we understand the importance of readability. However, the majority of the abbreviations are commonly used in the literature, which results in a more efficient presentation of the material.

Regarding the structure of the manuscript, we have carefully considered your suggestion to separate the results and discussion sections. However, we believe that a combined results and discussion section best serves the purpose of this article, providing a seamless flow of information and aiding in the visualization of data. We believe that in this case separating the results and discussion would result in too much repetition of the results.

Once again, we appreciate your thorough review and will make sure to address each concern diligently.

**Line 23: delete additional ‘the’**

AU: Thanks for your suggestion.

**Line 25 - 26: This needs to be rephrased. I presume the 95 samples were for development and the 23 samples were for validation? The sentence should reflect this, currently states development for both.**

AU: Thank you for your suggestion. We have rephrased the respective sentences to accurately reflect the sample allocation. Out of a total of 94 collected samples, 94 were utilized for developing the composition models. Within this dataset, a subset of 23 samples was specifically used for the *in situ* degradability assessment. Please check lines 24-28.

**Line 27: What are the ‘typical analytical methods’?**

AU: Thank you for pointing that out. In lines 26-27, I have rephrased it to “wet chemistry methods”, that refers to analytical techniques that are routinely utilized in animal nutrition laboratories. These methods are commonly employed for sample analysis and are recognized as standard practices in the field, ensuring accurate assessment of food composition and other relevant parameters.

**Line 94: Change ‘7’ to ‘seven’.**

AU: “7” was replaced by “seven”. The sentence now reads (Line 100).

**Line 101: Why was this the considering the appropriate forage: concentrate ratio and diet composition?**

Thank you for your valuable feedback regarding forage:concentrate ratio. We have made the necessary modifications to the text to address your question. The revised text now reads (Lines 106-111):

"The bulls were fed a 70:30 (DM basis) corn silage:concentrate (corn, soybean meal, wheat bran, urea, ammonium sulfate, salt, bicarbonate, and mineral) diet for ad libitum intake. The diet contained 12% crude protein (DM basis). The forage-to-concentrate ratio used was based on providing a balanced diet with diverse feed composition with enough fiber and energy to not compromise microbial growth. The animals were adapted to the experimental diet and conditions for 30 days before the *in situ* incubations."

**Line 344: It would be better to use different shapes as colour version will be online only, consider readers that may print the manuscript in black and white.**

Thank you for your suggestion. We appreciate your consideration for readers who may print the manuscript in black and white. We have modified the shapes in the figure and its accompanying legend to ensure clarity and differentiation even in black and white format. The sentence now reads (Line 359-360).

**Line 392: Replace the word ’globular’ with an appropriate word.**

AU: “globular” was replaced by “comprehensive”. The sentence now reads (Line 409).

**Table 1: Be consistent with the number of decimal places that data is presented to. Error should be presented as one more decimal place than mean (majority are okay but some values need to be corrected).**

Thank you for your I have revised the table to ensure consistency in the number of decimal places for the data. The error values now align with your suggestion of presenting one more decimal place than the mean values.
